# Advancing Insights into Probiotics during Vegetable Fermentation

**DOI:** 10.3390/foods12203789

**Published:** 2023-10-16

**Authors:** Yingzi Yuan, Yutong Yang, Lele Xiao, Lingbo Qu, Xiaoling Zhang, Yongjun Wei

**Affiliations:** 1 Laboratory of Synthetic Biology, School of Pharmaceutical Sciences, Zhengzhou University, Zhengzhou 450001, Chinaxiaolele22@outlook.com (L.X.);; 2 Food Laboratory of Zhongyuan, School of Pharmaceutical Sciences, Zhengzhou University, Zhengzhou 450001, China

**Keywords:** fermented vegetables, traditional fermentation, probiotics, lactic acid bacteria, microbiota

## Abstract

Fermented vegetables have a long history and are enjoyed worldwide for their unique flavors and health benefits. The process of fermentation improves the nutritional value, taste, and shelf life of foods. Microorganisms play a crucial role in this process through the production of metabolites. The flavors of fermented vegetables are closely related to the evaluation and succession of microbiota. Lactic acid bacteria (LABs) are typically the dominant bacteria in fermented vegetables, and they help inhibit the growth of spoilage bacteria and maintain a healthy gut microbiota in humans. However, homemade and small-scale artisanal products rely on spontaneous fermentation using bacteria naturally present on fresh vegetables or from aged brine, which may introduce external microorganisms and lead to spoilage and substandard products. Hence, understanding the role of LABs and other probiotics in maintaining the quality and safety of fermented vegetables is essential. Additionally, selecting probiotic fermentation microbiota and isolating beneficial probiotics from fermented vegetables can facilitate the use of safe and healthy starter cultures for large-scale industrial production. This review provides insights into the traditional fermentation process of making fermented vegetables, explains the mechanisms involved, and discusses the use of modern microbiome technologies to regulate fermentation microorganisms and create probiotic fermentation microbiota for the production of highly effective, wholesome, safe, and healthy fermented vegetable foods.

## 1. Introduction to Fermented Vegetable Foods

Fermented vegetables are popular traditional foods known for their unique flavors and health benefits [1]. The flavors of fermented vegetables can vary based on different vegetable materials and fermentation techniques used. A wide variety of fresh vegetables, such as cabbages, carrots, beets, cucumbers, celery, peppers, and green beans, can be used in the production of fermented vegetables [2]. Sauerkraut and pickles are the main types of fermented vegetables. Numerous studies have highlighted the health benefits of fermented vegetables, which provide nutritional products including vitamins, antioxidants, proteins, carbohydrates, and exopolysaccharides. Moreover, they exhibit anti-inflammatory, immunomodulatory, and gut-health-promoting properties [3,4,5,6,7].

The microbial metabolites produced during vegetable fermentation are essential in the formation of flavors. Microorganisms convert the fermentable substrates, mainly carbohydrates and proteins, into biologically active metabolites, such as short-chain fatty acids (SCFAs), sugar, organic acids, free amino acids (FAA), and volatiles. These metabolites contribute to the attractive flavors and desirable nutritional values of fermented vegetables [8,9]. Each type of fermented vegetables generally harbors a distinct population of microorganisms. Probiotics, especially lactic acid bacteria (LABs), are essential in fermented vegetables, for they optimize flavor characteristics, produce beneficial metabolites, inhibit undesirable microorganisms, and reduce harmful compounds [10,11]. LABs, known for their strong lactic acid production ability, become the dominant contributors during the later stage of vegetable fermentation [2,12,13].

Considering the contributions of vegetable fermentation and the metabolic activities of probiotic communities, fermented vegetables not only provide nutrition and appetizing healthy foods for humans but also offer various functional benefits. These benefits include antioxidant properties, cholesterol-lowering effects, and modulation of the gut microbiome [14,15,16,17,18]. Thus, it is critical to understand the interplay between the microbiota and physicochemical indices of fermented vegetables, in order to enhance the quality and safety of traditional fermented vegetable products.

This review provides an overview of the fermentation process and the mechanisms involved in traditional fermented vegetable production. It further discusses strategies for building a highly effective and healthy vegetable fermentation process with designed probiotics. These strategies include the application of biotechnologies to finely regulate probiotic activities, as well as the isolation and engineering of specific probiotic strains from fermented vegetables to optimize the fermentation process.

## 2. Traditional Vegetable Fermentation

**Figure 1:**
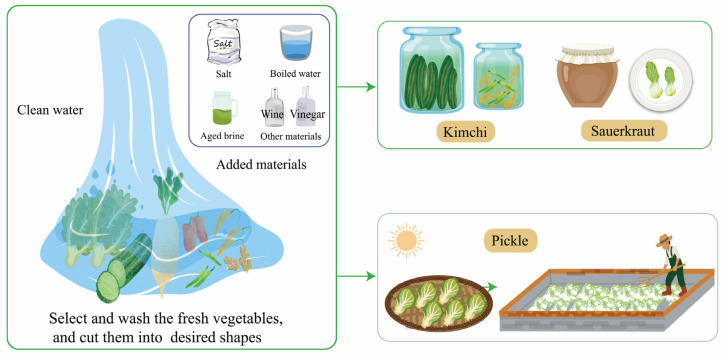
The production process of traditional fermented vegetables.

Due to different vegetable materials, environments, and production processes, traditional fermented vegetables can be roughly divided into sauerkraut and pickles. The cabbage is completely submerged in the brine containing a proper amount of salt, and the sauerkraut fermentation process occurs naturally [19]. Kimchi, similar to sauerkraut, includes a wider range of vegetables such as cabbages, peppers, daikon, long beans, garlic, and ginger. To shorten the fermentation process and improve fermentation quality, some vegetables would be fermented in salted brine in a certain fermentation process, with a circulating pump to maintain homogeneity [20]. Sauerkraut is compressed during fermentation and has a lower salt concentration compared to kimchi. Pickles are made by dehydrating various fresh vegetables under the sun for several hours. The semi-dried vegetables are then mixed with salt and placed in jars with water for spontaneous fermentation [2] (Figure 1).

### 2.1. The Fermentation Process of Traditional Fermented Vegetables

The traditional fermentation process involves several steps [21]. Firstly, fresh vegetables are selected and washed. Any rotten and moldy parts are removed to prevent fermentation issues and ensure food safety. Secondly, the vegetables are cut into desired shapes to facilitate the pickling process. Lastly, the vegetables are placed in a container, and salt water or aged brine is added. They are then marinated under specific conditions for a few days.

During fermentation, the conditions, such as temperature, salt concentration, and fermentation time, are adjusted based on the specific vegetables being fermented. For example, white cabbages cultivated in South Tyrol are fermented for 42 days at 15 °C with 1.3–1.4% salt according to the traditional procedure [19]. Peppers, on the other hand, undergo spontaneous fermentation in sterile water (boiled water) with 0.5–3.5% salt for about 10 days [22]. Temperature plays a significant role in the growth of LABs and their acid production ability, which affects the pH and fermentation speed [23]. Salt concentration and pre-salting have a crucial impact on the quality of fermented vegetables [24], influencing microbial diversity and volatile compounds. Certain bacterial species, such as *Pediococcus*, *Leuconostoc*, *Weissella*, *Sporobolomyces*, *Azospirillum*, *Klebsiella*, *Acinetobacter*, and *Cladosporium*, have a close correlation with salt concentration during vegetable fermentation. However, *Lactobacillus* is not affected by different salt additions (0%, 3%, 6%, and 9%) and tends to dominate the fermentation process, especially in the later phase of fermentation [24]. In some vegetable fermentation procedures, aged brine is used while others simply use salt water. Aged brine can have a significant impact on microbial growth at the beginning of fermentation and contribute to the production of more aromatic compounds, higher organic acid content, and a lower pH value [25]. However, the fermentation functions of certain species in aged brine are not well understood, which limits our ability to regulate microorganisms and predict the quality of fermented vegetables.

Traditional vegetable fermentation primarily relies on the naturally occurring bacteria and fungi presenting on the fresh vegetables or in the food processing environment. Based on metagenomic screening, highly diverse microorganisms have been revealed, which include fungi and bacteria [26]. The fungi have been reported to play a significant role in flavor formation in fermented chili pepper [27], broad bean paste [28], and fermented Da-jiang [29]. Diverse fungi, such as *Cladosporium*, *Candida*, *Aspergillus*, *Pichia*, *Sporobolomyces*, *Debaromyces*, *Psathyrella*, and *Debaryomyces hansenii*, have been observed in various fermented vegetables [4,24,30,31,32]. Both the bacterial and fungal species are strongly associated with increased concentrations of organic acids, amino acids, biogenic amines, and volatiles [31]. However, yeasts and molds can produce metabolites that result in undesirable taste and smell, and they can inhibit lactic acid production [33]. Bacteria, especially *Lactobacillus*, make up the majority of the microbiota involved in vegetable fermentation and gradually become dominant in the later stage of fermentation [34].

### 2.2. Insight into the Microbiota of Traditional Fermented Vegetables

The physicochemical attributes of fermented vegetables, such as pH, acidity, nitrite, texture, and color, undergo constant changes during the fermentation process. Similarly, the flavor properties, such as sugar, organic acids, esters, terpenes, alcohols, and phenols, increase significantly [19,31]. As a result, the growth of certain microbial species was inhibited, leading to a decrease in the abundance of the initially dominant genera [35]. The *Lactobacillus* tends to dominate the fermented microbiota in the later stage of fermentation. In Jiangxi yancai, Sichuan paocai, and Dongbei suancai, the abundance of *Levilactobacillus* can be as high as 73.7% [2].

The dominant bacteria can vary depending on the type of vegetable, the production process, and the fermentation conditions. For instance, pickled chili pepper, a traditional Chinese fermented vegetable, is fermented with aged brine in an open-air pickling tank for at least 3 months in Jianshui city, China. In this situation, *Lactobacillus* remains the main genus throughout the entire fermentation process [34]. In contrast, the spontaneous fermentation of pickled pepper in North China involves sterile water (boiled water) with salt in an oxygen-free environment, where the composition of *Lactobacillus* is 49.6% [22].

The flavor profiles of different fermented vegetables are closely related to specific bacterial species, which exhibit great diversity [36]. The microorganisms that are most adaptable to the fermentation process are responsible for producing the flavors [37]. Certain core bacterial species within the microbiota are believed to be the key contributors to the flavors. For example, *Lactobacillus alimentarius* may contribute fruity, sweet, and floral odors to pickled chayote, while other *Lactobacillus* species, *Lactobacillus futsaii* and *Lactobacillus paralimentarius*, in particular, generate a sour taste to the products [35]. The abundance of *Lactobacillus alimentarius* increased significantly during the fermentation of pickled chayote, suggesting it is essential [35]. Additionally, *Secundilactobacillus malefermentans* has been identified as a keystone species in the fermentation microbiota, especially during the last weeks of fermentation [19]. In pickled chili pepper, *Lactobacillus versmoldensis* and *Lactobacillus brevis* were identified as keystone microorganisms highly related to flavor production [34]. These bacterial species could potentially serve as starter cultures to optimize fermentation processes and achieve fermented vegetables with improved and standardized nutritional and sensory characteristics.

#### LABs during Vegetable Fermentation

LABs are Gram-positive and catalase-negative microorganisms. They are acid-resistant, facultatively anaerobic, morphologically globular, or rod-shaped, and they do not form spores [38,39]. LABs are widely distributed in various sources, such as food, plants, soil, animals, and the human body. The genera of LABs include *Lactobacillus*, *Lactococcus, Pediococcus*, *Enterococcus*, *Streptococcus*, *Leuconostoc*, *Weissella*, and others [14,38,40,41]. While there is a considerable diversity of LABs, food is one of the most common sources. Fermented foods, such as fermented dairy products, fermented vegetables, fermented meat, and fermented grains, are particularly rich in LABs. Fermented vegetables, being a common and easily accessible product, are often used as raw materials for isolating LABs. Certain isolated LAB strains derived from fermented vegetables exhibit probiotic effects. For example, *Lactiplantibacillus pentosus* CF2-10N isolated from fermented Aloreña green table olives can produce exopolysaccharide and vitamins and demonstrate an immunomodulatory effect [42]. *W. koreensis* SK isolated from kimchi can produce ornithine, which has an anti-obesity effect [43]. *Lactiplantibacillus plantarum* LRCC5314, isolated from kimchi, has anti-inflammatory effects [44]. *L. pentosus* LPG1 isolated from edible olives can produce bacteriocin and exopolysaccharides, exhibits an anti-inflammatory effect, reduces cholesterol levels, and inhibits food-borne pathogens [45].

LABs have a range of functions, including immune regulation, intestinal improvement, inhibition of food-borne pathogens, and an anti-inflammatory function [38,46,47]. Therefore, LABs have applications in various fields, including food and medical treatment, with the production of fermented food being one of the most common uses. During the fermentation process, LABs produce various metabolites, primarily lactic acid, bacterin, amino acids, exopolysaccharides, ornithine, aldehydes, and esters. When LABs are used as starter cultures for fermenting vegetables, they contribute to the production of beneficial fermented vegetable products. For example, inoculating cabbage with *L. plantarum* CGMCC No. 20193 and *P. pentosaceus* CGMCC No. 20192 increases the content of amino acids and other beneficial substances while reducing the nitrite content, making the product healthier [48]. Fermentation with *L. plantarum* ZJ316 increases the content of mustard, aldehydes, and esters, as well as the number of probiotics [49]. *Lactobacillus paracasei* HD1.7 produces bacteriocin Paracin 1.7, which helps inhibit the growth of pathogenic bacteria during cabbage fermentation [50]. Fermented carrot from *L. plantarum* 299v has been shown to have potential benefits in treating and preventing obesity and type 2 diabetes [51].

### 2.3. The Mechanisms of Traditional Fermented Vegetables

**Figure 2:**
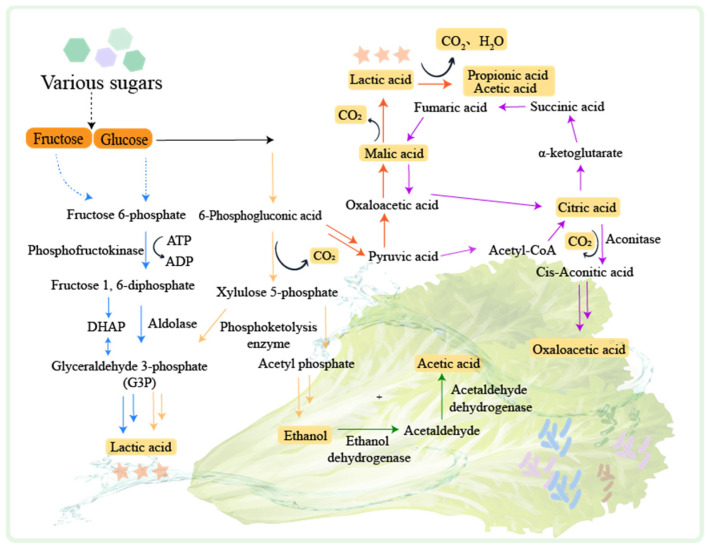
The mechanisms of fermented vegetables. PTS: Phosphoenolpyruvate-dependent sugar phosphotransferase system. The homotypic lactic fermentation pathway is indicated by the blue arrow. The heterotypic lactic fermentation pathway is indicated by the yellow arrow. The malate metabolic pathway is indicated by the red arrow. The citrate metabolic pathway is indicated by the purple arrow. The ethanol metabolic pathway is indicated by the green arrow.

LABs play a leading role in fermented vegetable production. LABs can be classified according to their distribution environment of homofermenters, heterofermenters, and facultative fermenters [52]. Lactic fermentation is the primary process during the fermentation of vegetables and can be divided into homotypic fermentation and heterotypic fermentation [53] (Figure 2).

In hetero-lactic fermentation, glucose is converted to 6-phosphogluconate by hexokinase and glucose dehydrogenase [54], and then to ribulose-5-phosphate by 6-phosphogluconate dehydrogenase. Xylulose-5-phosphate is generated by the epimerization of ribulose-3-epimerase, which is then decomposed to acetyl phosphate and glyceraldehyde-3-phosphate. Acetyl phosphate is converted to acetyl-CoA by phosphotransacetylase, which is then processed by aldehyde dehydrogenase and alcohol dehydrogenase to produce ethanol [55]. Glyceraldehyde-3-phosphate undergoes glycolysis to generate pyruvate, which is converted to lactate by lactate dehydrogenase. In homo-lactic fermentation, glucose is catalyzed by hexokinase and phosphohexose isomerase to produce fructose 6-phosphate and then decomposed into dihydroxyacetone phosphate (DHAP) and glyceraldehyde-3-phosphate (G3P) by aldolase. G3P undergoes a series of reactions and is finally converted to pyruvate which is reduced to lactate [53], and lactic acid can be further converted to propionic acid and acetic acid by *Propionibacterium* [56].

Apart from lactic fermentation, vegetable fermentation also involves the production of ethanol, citric acid, and malic acid [52]. Ethanol can be converted to acetic acid by alcohol dehydrogenase and acetaldehyde dehydrogenase [57]. Citric acid generation is usually carried out in the presence of *Aspergillus niger*. The pyruvate generated by glycolysis is converted to acetyl-CoA and oxaloacetate. Oxaloacetate is reduced to malate, which participates in the tricarboxylic acid cycle to produce citrate. Citric acid can be converted to oxoacetic acid through the tricarboxylic acid cycle [58], and malic acid generated in this process can be fermented by *Oenococcus oeni* to produce lactic acid [59].

During vegetable fermentation, hetero-lactic fermentation is dominant at the early stage [60]. This process is carried out by microorganisms such as *Bifidobacterium* spp., *Cryptococcus* spp., and certain *Lactobacillus* such as *Lactobacillus brevis* and *Lactobacillus buchneri* [61]. Hetero-lactic fermentation reduces the pH of the environment, creating an unfavorable condition for the activity of LABs. At the middle stage of fermentation, homo-lactic fermentation occurs. This process is mainly performed by *Lactobacillus delbrueckii*, *L. plantarum*, and *Streptococcus* spp. These bacteria can convert more than 80% of glucose to lactic acid. At the later stage of fermentation, various compounds are produced by LAB fermentation. These compounds include organic acids, glycolyl, acetoin, acetic acid, ethanol, short peptides, and amino acids. They contribute to the flavor of fermented vegetables. At the later stage of vegetable fermentation, alcoholic and acetic fermentation processes also take place. The pH of the environment decreases, which slows down the LAB growth. In the meanwhile, the yeast and acetic acid bacteria become dominant. The yeast converts the sugars in vegetables into ethanol through alcohol fermentation. The acetic acid bacteria then convert the ethanol into acetic acid. Additionally, the acetic acid combines with alcohols to form esters, which further enhances the flavor of fermented vegetables.

## 3. The Effects of Probiotics on Fermented Vegetables

In recent times, probiotic starter cultures have become increasingly popular as essential contributors to vegetable fermentation due to their numerous benefits. These benefits include reducing harmful metabolic products, inhibiting pathogenic bacteria, and enhancing therapeutic effects. Probiotics play a significant role in promoting carbohydrate, amino acid, and nucleotide metabolisms, as well as reinforcing the biosynthesis of vitamins and bacteriocin. Moreover, they contribute to the generation of therapeutic products [62]. For example, a formulation that includes *Lactobacillus acidophilus* GL A-14, *Lactobacillus rhamnosus* HN001, and bovine lactoferrin can be used as an adjuvant therapy along with topical clotrimazole to treat vulvovaginal candidiasis. Individuals who used the adjuvant therapy experienced better improvement [63]. Additionally, in an in vivo study, Golden Syrian hamsters were orally administered a vaccine containing *L. casei*, and the result showed that the vaccine exhibited significant advantages when compared to the traditional vaccine [64]. *L. plantarum* CQPC02 isolated from the Sichuan pickle showed significant anti-fatigue and anti-oxidation effects in fatigue mice models, suggesting that it can be a potential microbiological therapeutic agent [65]. Organic acids produced by probiotics act as natural preservatives, prolonging the shelf life of fermented products and influencing flavor formation.

Probiotics possess metabolic capacity for various carbohydrates, including glucose, fructose, xylose, ribose, trehalose, maltose, and sucrose, and organic acids, including citrate, malate, and fumarate. The presence of fructose has been found to increase the expression of certain genes related to these metabolisms [66]. Phenolic derivatives derived from the microbial metabolism of probiotics significantly affect the sensory and health-promoting features of fermented vegetables. *Lactobacilli* and *Pediococcus* spp. are prominent producers of lactic acid and exhibit a high metabolic potential for the bioconversion of phenolics [19]. Bacteriocins produced by LABs have potential use as natural food preservatives due to their excellent antibacterial effects [38]. For instance, *L. plantarum* RUB1 has been found to produce a class IIb bacteriocin with strong antibacterial activity [67]. *L. paracasei* LS-6, which was isolated from a traditional fermented yogurt in Yunnan, China, can produce a bacteriocin LSX01 that exhibits activity against *Staphylococcus aureus* [68]. Additionally, nine strains isolated from home-made fermented vegetables from Northwest Bulgaria were found to be bacteriocin producers. These strains could potentially be utilized as starter cultures, reducing the need for chemical preservative additives in fermented vegetables [69]. Probiotics also have the ability to produce γ-aminobutyric acid (GABA), a major inhibitory neurotransmitter in the central nervous system. In addition, GABA exhibits anti-anxiety and tranquilizing effects [70,71]. Certain strains, such as *Latilactobacillus curvatus* K285 isolated from gat-kimchi [72], *L. pentosus* 9D3 isolated from Thai pickled weed [73], *L. plantarum* KB1253 [74], and *Companilactobacillus allii* WiKim39 and *Lactococcus lactis* WiKim0124 isolated from kimchi [75] have demonstrated a strong ability to produce GABA, making them potential starter cultures for producing functional foods. Mannitol, a 6-carbon sugar alcohol naturally present in microorganisms and plants, is slowly absorbed in intestinal tracts and does not increase blood sugar levels [70,76]. Strains such as *Leuconostoc mesenteroides* SKP88 and *Leuconostoc citreum* SKP92 isolated from pa (green onion)-kimchi can convert fructose to mannitol [76]. Ornithine, a non-proteinogenic amino acid converted from arginine, offers various functions such as anti-obesity properties, muscle growth promotion, anti-fatigue effects, and cirrhosis treatment [70]. *Weissella koreensis* DB1 can produce 15,059.65 mg/L ornithine. Safety evaluations have shown that it poses no health risk and can be used in fermented foods [77]. Fermented vegetables are rich in vitamins, which are essential for the proper functioning of the human body. The fermentation process helps retain the vitamin content from the raw materials [78]. In particular, co-fermentation of *Pediococcus pentosaceus* AL and *Cyberlindnera rhodanensis* J52 significantly increases the vitamin C concentration in fermented capsicum [79]. Additionally, certain LABs have the ability to produce vitamins. For example, *Lactobacillus reuteri* F2 has shown a strong ability for extracellular vitamin B12 production [80]. Therefore, fermenting vegetables with probiotic strains can serve as an effective method to enhance their beneficial properties.

Probiotics, such as LABs, have the ability to inhibit the growth of pathogenic bacteria and reduce the negative effects of harmful substances due to high lactic acid generation capacity, thereby improving the quality and safety of fermented products [49,81]. LABs, such as *L. brevis* and *L. plantarum* ZJ316, can inhibit the growth of *Ralstonia* spp., *Pseudomonas*, *Proteus*, and *Enterobacter* in pickled chili pepper and pickled mustard [34,49]. Functional foods rich in probiotic LABs have the potential to combat accidentally ingested pesticides in the gastrointestinal tract directly [8]. The high lactic acid generation capacity of LABs, such as *L. plantarum* ZJ316, contributes to the reduction in nitrite residual levels [49]. Deltamethrin, dimethoate, and imidacloprid are common pesticides used during olive growth, which are harmful to human health. Natural black olive fermented with *L. plantarum* strains 112 and 123 showed higher degradation of these substances compared to crude olives [82] (Table 1). The consumption of probiotics in fermented vegetables can have a significant impact on the composition of gut microbiota. When mice were fed with green loofah fermented with *L. plantarum* Uruma-SU4, the level of *Lactobacillus johnsonii*, which is the predominant LAB in mice gut microbiota, was increased [73]. The Firmicutes/Bacteroidetes ratio (F/B ratio) can reflect the health of the gut microbiota, with a higher F/B ratio suggesting low gut microbiota diversity [74]. *L. pentosus* P2020 derived from the Chinese pickle can significantly lower the F/B ratio and restore the gut microbiota, thereby protecting against the development of hyperuricemia [75]. Similarly, *L. plantarum* TWK10 isolated from Taiwan pickled cabbage has been found to reduce the F/B ratio in aging mice and modulate the imbalance of gut microbiota, thereby attenuating aging-related disorders [76]. These findings suggest that vegetables fermented with probiotics have the potential to become valuable contributors to therapeutic interventions aimed at restoring gut microbiota.

### 3.1. Application of Probiotic Starter Cultures in Vegetable Fermentation

Spontaneous fermentation often leads to poor-quality products, as they are susceptible to contamination by spoilage microorganisms and pathogenic bacteria, which poses a challenge for industrial production [97]. Additionally, there is a growing consumer demand for fresh-tasting, nutritionally rich, and health-promoting foods with pleasing sensory properties. To meet these demands, specific microbial species with desired properties can be isolated from fermented vegetables to be used as starter cultures in the production of functional food [93]. The use of probiotic starter cultures in fermentation offers effective approaches to standardize product quality, ensure safety, and optimize the benefits of the final products [98].

Probiotic starter cultures, mainly consisting of LABs, can be used in vegetable fermentation either as single-strain or mixed-strain cultures, depending on the specific fermented vegetable products. The selection of starter cultures significantly influences the physicochemical properties and aromatic qualities of the fermented vegetables [98]. Fermentation with a single bacterial species can accelerate the acidification process, resulting in faster conversion of fermented vegetables, reduced commercial losses, and lower production costs [20,81]. Fermentation with mixed probiotic starter cultures can enhance the fermentation abilities and shorten the maturation period. The mixed fermentation microbiota consists of dominant microorganisms, and the flavors of fermented vegetables are primarily derived from these dominant species. Kimchi inoculated with different starters exhibit high ratios of *Leuconostoc*, *L. plantarum*, and *L. brevis* [99]. A high inoculum of *L. plantarum* and *P. pentosaceus* strains (NPL 1258 and NPL 1259) has been found to effectively control the quality of fermented cucumbers [94]. Furthermore, the different mixing ratios of starter cultures lead to distinct metabolites in fermented vegetables. A high-level *L. mesenteroides* inoculation exhibits hetero-fermentative characteristics, resulting in higher mannitol and acetic acid levels but lower lactic acid levels compared to high-level homo-fermentative *L. sakei* inoculation [100].

By increasing the population of LABs and decreasing the undesirable microorganisms, probiotic starter cultures contribute to a shorter fermentation cycle and a reduction in the population of pathogenic organisms, thereby improving the safety and quality of fermented vegetables. Heterofermentative LABs can accelerate the growth of homofermentative LABs, leading to a rapid decrease in pH value [92]. Additionally, *L. plantarum* exhibits strong acid tolerance and performs well in the presence of *Leu. mesenteroides*. Therefore, the starter culture composed of *L. plantarum* and *Leu. mesenteroides* plays a significant role in producing the distinctive flavor of northeast sauerkraut [97].

### 3.2. Design of Robust, Stable, and Predictable Probiotic Microbiota

**Figure 3:**
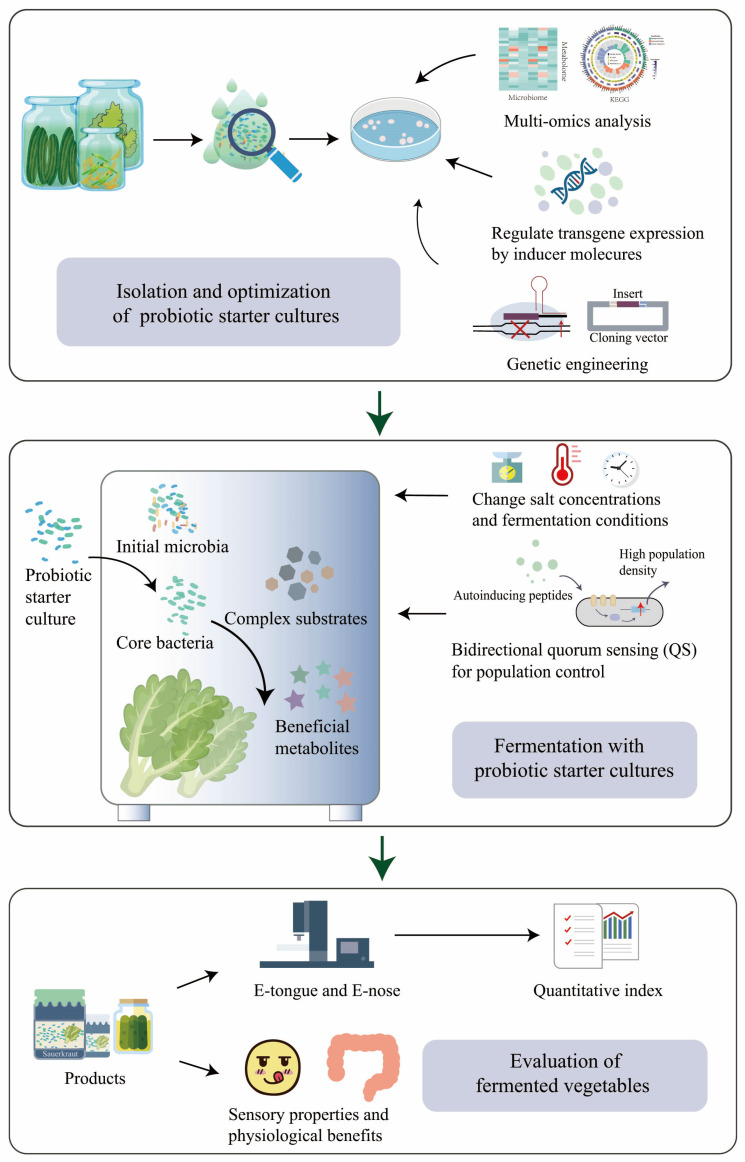
Design of robust, stable, and predictable vegetable fermentation with probiotic starter cultures.

Designing a robust, stable, and predictable probiotic microbiota is crucial for ensuring the quality of fermented vegetable products. The presence of an adequate number of probiotics is essential for their health benefits, with a minimum of 10^6^ CFU/mL being necessary [101]. To achieve this, supplementing the fermentation process with dietary fiber, such as cellulose or inulin, before fermentation, not only generates prebiotic carbohydrates but also creates a favorable growth environment for probiotic strains, thereby improving their viability in fermented vegetable foods [94,102,103] (Figure 3). Furthermore, establishing a microbial collection that encompasses various microbial strains and their corresponding genomic information can offer a valuable resource for developing effective probiotic strains and providing strains for designing probiotic microbiota [104,105].

Probiotics are living microorganisms that confer beneficial effects on the host [106]. By establishing communication systems through intercellular signaling and facilitating co-culture to create interdependent networks of microorganisms, it is possible to enhance the gastrointestinal environment tolerance of probiotics and enhance the beneficial properties of fermented vegetables [107,108]. Quorum sensing (QS) is a biological communication system that regulates various physiological and biochemical functions, including gene expression, biofilm formation, and bacteriocin production. This system operates through the use of specific signal molecules called autoinducers [109,110]. For example, under co-culture conditions, *L. acidophilus* can enhance its intestinal adhesion ability and promote the growth of other strains in the starter culture by the autoinducer-2 QS system [111] (Figure 3). As for LABs and *Bifidobacteria*, QS is essential for their resistance to harsh conditions and biological function regulation [112].

To improve the characteristics of fermented vegetables and achieve cost-effective industrial production with high quality, various strategies have been implemented. For example, the biotechnological strategy of roseoflavin induction was used to increase riboflavin content by 10 times in *L. plantarum* RYG-YYG-9049-M10 [113]. Additionally, the characterization of the pan-genome of *L. sakei* allows for the exploitation of its genomic diversity and the identification of marker genes, enabling the establishment of starter strain sets with complementary metabolic traits [114]. In the case of *L. plantarum* CGMCC 1.2437T, it has the capability to produce GABA. By using L-monosodium glutamate as a single inducing factor, GABA synthesis is facilitated while degradation is inhibited [115]. Another strategy involves co-culturing *L. acidophilus* and *Bacillus subtilis*, which can enhance the production of total SCFAs [116].

The advancements in metabolic engineering and synthetic biology have enabled wild-type microorganisms to acquire additional functionalities [117]. For instance, engineered yeast can now synthesize diverse plant natural products [118,119,120], while probiotic strains can be engineered to possess enhanced functional properties [121]. Furthermore, the design–build–test–learn cycle of synthetic biology has been employed to construct robust and stable probiotic microbiota [122]. These developments have great potential to improve the process of fermented vegetable production and offer ideal probiotic starter cultures for vegetable fermentation.

## 4. Expectations for Modern Vegetable Fermentation

Currently, multi-omics analyses based on gas chromatography mass spectrometry, next-generation sequencing, and other advanced technologies can be applied to investigate the intricate association and interactions between microorganisms and their metabolic products [123]. The multi-omics approach has revealed the functions of diverse microbiota [124,125,126,127]. Metagenomics and metabonomics can be applied to investigate the microbial dynamics of kimchi after inoculating LAB starter combinations [128]. Metabolomics analysis and metagenomic sequencing revealed that the consumption of Zhàcài potentially prevented high-fat-induced dyslipidemia through gut microbiota [129]. Additionally, multi-omics analyses allow for the identification of bacterial species with outstanding fermentation properties, facilitating the evaluation of individual strains as potential starter cultures [130]. For instance, through the metagenomic sequencing of wheat-based thin stillage, a predominant *Lactobacillus* species was discovered, providing a cost-effective means of producing high-quality protein and commercial ingredients [131]. To achieve large-scale fermentation levels and meet the demands of high-quality and cost-effective industrial production, it is crucial to enhance the accumulation of nutritional products and improve probiotic tolerance toward challenging environments [132]. Therefore, vegetables fermented with designed probiotic starter cultures can offer consumers more desirable health benefits and improved flavors.

The composition of metabolites in fermented vegetables reflects the overall phenotype of the entire microbiota [133]. Changes in metabolites serve as key indicators of the activity of specific bacteria during sauerkraut fermentation [134]. Additionally, the population of distinct microbial species acts as an indicator for evaluating the quality of fermented vegetables [135,136]. Improving the vegetable fermentation process can be achieved by establishing a more controllable fermentation platform and quantifying fermentation indices using techniques such as e-tongue and e-nose [137] (Figure 3). E-tongue and e-nose technologies offer digital methods to simulate human taste and olfaction, enabling rapid and accurate evaluation of food flavors [138] (Figure 3). For example, in the flavor characterization of traditional Chinese fermented soybean paste, the combination of intelligent sensing technologies and chemometrics exhibited high discriminant accuracy [139]. By using an e-nose, e-tongue, and sensory evaluation, LAB fermentation significantly reduced the signal association with undesired tastes in a blended edible rose and shiitake beverage [140] (Figure 3). The process of vegetable fermentation is complex, and understanding its mechanism remains challenging. The selection of appropriate starter strains, bacterial interactions, and optimization strategies are still under investigation. Further studies are needed to reveal the metabolic activity and interactions of microorganisms in vegetable fermentation probiotic starter cultures.

## 5. Conclusions

Fermented vegetables are known for their abundant bioactive compounds and probiotics, which provide various beneficial biological activities, including anti-inflammatory effects and immunomodulation [3,141,142]. The core microorganisms, often *Lacticaseibacillus*, are essential in shaping the characteristics and quality of fermented vegetables. Understanding the relationship between the microbiota and metabolites provides valuable insights into the development of flavors and qualities in fermented vegetables. In the future, utilizing probiotic starters generated with advanced synthetic biology tools instead of relying on spontaneous fermentation can yield several advantages. These include shortening the fermentation period, accelerating the production of metabolites, increasing the abundance of potential probiotics, and reducing the presence of pathogenic bacteria. These advancements can help in providing consumers with healthier fermented vegetables that promote overall well-being.

## Figures and Tables

**Table 1:** The metabolic products and health benefits of microbial strains in fermented vegetables.

Microbial Strains	Isolation Source	Metabolic Products	Health Benefits	Study Type	Ref.
*Leu. mesenteroides/L. plantarum*	Kimchi	Benzyl isothiocyanate, indole compounds, thiocyanate, b-sitosterol, and dietary fiber	Anticancer, anti-atherosclerotic, and cholesterol-lowering	In vivo	[83]
*L. plantarum* JS19	Shaanxi Jiang-shui		Ameliorate inflammatory bowel disease	In vivo	[84]
*W. paramesenteroides*	Yan-dong-gua	Organic acids, hydrogen peroxide, and bacteriocins	Antibacterial	In vitro	[85]
*B. subtilis natto*	Natto	Nattokinase, dipicolinic acid, Menaquinone-7	Anti-thrombotic, promote bone health	Clinical trial	[86]
*L. plantarum/Lev. Brevis/Leu. fallax*	Fermented cucumber	GABA	Antihypertensive, antidepressant, and anticancer	In vitro	[87]
*L. plantarum/L. pentosus*	Table olives	Bioactive compounds, dietary fibers, fatty acids, antioxidants	Antioxidant activity	In vivo	[88]
*L. pentosus* LPG1	Table olive	Bacteriocin, exopolysaccharides	Anti-inflammatory, reduce cholesterol levels, and inhibit food-borne pathogens	Clinical trial	[45,89]
*L. fermentum* SHY10	Chinese pickles	Antibacterial peptides	Antibacterial	In vitro	[90,91]
*L. pentosus* CF2-10N	Fermented Aloreña green table olives	Vitamin, exopolysaccharide	Immunomodulation	In vitro	[42]
*L. plantarum* Uruma-SU4	Fermented loofah		Bile acid-lowering	In vivo	[92]
*L. plantarum* LRCC5314	Kimchi		Anti-inflammatory, inhibition of adipogenesis	In vitro	[44]
*Lactococcus lactis*/*Weissella cibaria*	Fermented beetroot	Niacin, riboflavin	Antagonistic properties against pathogenic bacteria	In vitro	[93]
*L. plantarum* NPL 1258 *P. pentosaceus* NPL 1264	Fermented cucumber	Extracellular polymeric substances (EPS)	Shorten the fermentation cycle and reduce pathogenic organism populations	In vitro	[94]
*L. pentosus* P2020	Chinese pickle		Lower serum uric acid	In vivo	[95]
*L. plantarum* TWK10	Pickled cabbage		Attenuate aging-related disorders	In vivo	[96]
